# Pro-Inflammatory Cytokines Expressed During the Initial Phases of Osseointegration: A Systematic Review

**DOI:** 10.3390/jcm13237247

**Published:** 2024-11-28

**Authors:** Matt Baker, Daniel Fernandes, Carlos Marcelo S. Figueredo

**Affiliations:** 1 School of Medicine and Dentistry, Griffith University, Queensland 4222, Australia; matt.baker2@griffithuni.edu.au (M.B.); d.fernandes@griffith.edu.au (D.F.); 2 Division of Oral Diseases, Department of Dental Medicine, Karolinska Institute, 171 77 Stockholm, Sweden

**Keywords:** dental implants, osseointegration, cytokines, systematic review, pro-inflammatory, early healing

## Abstract

**Background:** Identifying patients with a strong pro-inflammatory phenotype may allow clinicians to underpin high-risk individuals based on early inflammatory marker profiles and to personalize approaches to preventative treatments. **Aim:** The objective of this systematic review is to synthesize the results of previous studies on osseointegration to show which pro-inflammatory cytokines and chemokines have been detected and quantified during the initial phase of osseointegration. **Material and methods:** PubMed, Embase, Scopus, ISI Web of Science, and Cochrane Library were searched for articles published until August 2024. A descriptive summary was produced to explain study variations, including patients’ characteristics and results. The methodological quality of each included study was assessed based on Downs and Black’s checklist. **Results:** 30 studies were selected for inclusion. In total, 710 patients received 1329 implants (an average of 1.87 implants per participant). A total of 32 biomarkers were analyzed. The overall trend observed in levels of pro-inflammatory cytokines and chemokines appears to be an early peak, followed by a progressive reduction in levels throughout the observation periods. **Conclusions:** The available evidence suggests that a strong expression of pro-inflammatory biomarkers is a feature of osseointegration, and an over- or underexpression of certain biomarkers could have an effect on early marginal bone levels. Several of these markers are mechanistically implicated with implant pathology; however, the prognostic value of early cytokine expression and correlation with long-term clinical outcomes requires further research.

## 1. Introduction

Surgical trauma to tissue during implant placement initiates a complex set of inflammation-related reactions, involving bone resorption, bone apposition, angiogenesis, and neurogenesis. This dynamic inflammatory process must be carefully regulated and liaised with cells from the immune system [1,2]. (Indeed, any implantable device triggers an immune response, whereupon the immune system can be either up- or downregulated, with wide repercussions on the healing process [1,3]). The success of implantation relies on the establishment of a successful structural and functional interface between the surface of a load-bearing implant and the surrounding living bone tissue, which is understood as osseointegration, which can be enhanced by surface modifications to achieve early osseointegration [4].

The inflammatory process during the early phases of healing following implant placement provides a unique window into immune function [2]. The immune cells from the peri-implant tissues play an important role in the local micro-environment, as osteoimmune modulation is a mandatory mechanism to be tailored to achieve a proper osseointegration [1,2,3]. The most relevant cells involved are osteocytes, osteoblasts, and ultimately osteoclasts, with most of their cross-talks mediated by signaling molecules such as cytokines and chemokines [5,6,7]. The detection of such signaling molecules in peri-implant crevicular fluid (PICF) and their utility in monitoring osseointegration has been documented in the literature [1,8,9,10].

PICF is a complex mixture of substances derived from serum and host tissues, as well as from oral bacteria. A study by Khoury et al. in 2008 [11] was among the first to examine the presence of inflammatory markers during the early stages of single-stage implant healing, detecting both IL-1β and IL-8 during the first week following implant placement. Gruber et al. [12] detected IL-1β in PICF in immediately placed dental implants as early as 1 day post-operatively in 2009, while Slotte et al. (2012) [13] examined levels of IL-1β and TNF-α from 2 days post-implant placement. Since these early studies, several other biomarkers have been investigated, including Interleukins-6, -8, and -17, as well as other markers, including RANKL [14,15,16]. Many of these markers serve multiple physiological functions; however, they all play key roles in the inflammatory healing cascade and coordinate an effective response to acute trauma and return to tissue homeostasis.

Although the expression of pro-inflammatory markers experiences qualitative and quantitative fluctuations among individuals [17] the maintenance of functional osseointegration is always required by an ongoing balance between bone resorption and apposition [18]. Whilst many of the biological complications associated with implants are a result of dysregulated bone homeostasis, it is of paramount importance to understand the presence and function of pro-inflammatory markers, which are possible targets across host-modulation strategies to optimize osseointegration. Moreover, identifying patients with a strong pro-inflammatory phenotype may also allow clinicians to underpin high-risk individuals based on early inflammatory marker profiles and personalize approaches to preventative treatments. Herein, we hypothesized that high expression of pro-inflammatory cytokines during the early stages of osseointegration could increase the risk of future implant failure.

Therefore, the objective of this systematic review is to synthesize the results of previous studies on osseointegration to show which pro-inflammatory cytokines and chemokines have been detected and quantified during the initial phase of osseointegration.

## 2. Materials and Methods

The reporting for this review adheres to the Preferred Reporting Items for Systematic Reviews and Meta-Analyses (PRISMA) statement guidelines.

### 2.1. Focus Question

“Which pro-inflammatory cytokines and chemokines are present in the Peri-Implant Crevicular Fluid from early healing until osseointegration following implant installation”?

### 2.2. Search Strategy

Electronic searches were conducted by two examiners (MB, CMFS). Medical Subject Headings (MeSHs) and relevant keywords were utilized on PubMed to access Medline. The search strategy was adapted for additional electronic databases, including Embase, Scopus, Cochrane Library, and Web of Science. The search was restricted to English-language articles published through August 2024. The search used combinations of terms as follows: (“Dental Implants” or “Dental implantation”) and (“Osseointegration” or “Osteogenesis” or “Bone Resorption”) and (“Biological Markers” or “Inflammatory Markers” or “Cytokines” or “Interleukins”). No date limit was applied. The Reference Lists from retrieved papers were hand-searched to identify additional eligible studies. Where inter-examiner disagreements about article inclusion were found, articles were discussed until a consensus was agreed upon.

### 2.3. Selection Criteria

Inclusion Criteria: (1) clinical trials with longitudinal outcomes involving participants who were partially or completely edentulous, receiving titanium dental implants placed as a single-stage procedure; (2) at least one PICF collection performed during osseointegration (up to 12 weeks post-surgery); (3) pro-inflammatory cytokine expression (level or concentration) analyzed during healing by any technique; (4) report written in English.

Exclusion Criteria: (1) literature reviews, case studies, or animal model studies; (2) biomarkers quantified from blood, saliva, or mucosal biopsy or punch specimens; (3) mini implants used for orthodontic anchorage or zirconia implants; (4) studies that performed simultaneous bone augmentation or guided bone-regeneration techniques; (5) PICF collected after osseointegration (12 weeks post-surgery or loading).

### 2.4. Data Synthesis

From each included paper, data were extracted and expressed in chronological order according to publication date. A descriptive summary was produced to explain study variations, including patients’ characteristics and results. Data elements extracted included the following: author names, publication year, study type, objectives, number of patients, number of implants, implant system, implant type, follow-up, outcome variables, bone sites, prosthetic treatment, use of antibiotics, implant loading, inflammatory markers, PICF analyses, biomarker concentration, significant biomarker results, and main findings.

### 2.5. Quality Assessment

Two reviewers assess the risk of bias in included trials independently. The methodological quality of each included study was assessed based on Downs and Black’s checklist [19], which consists of 27 items across 5 domains (reporting, external validity, bias, confounding, and power). Answers were scored either 0 or 1, except for one reporting item (scored 0–2) and statistical power (0–5). The checklist scores were grouped into the following four-level quality index [20]: ≤14, poor; 15–19, fair; 20–25 good; and 26–28, excellent. For studies that were single-arm in nature, a modified version of the Downs and Black’s checklist was used ().

## 3. Results

### 3.1. Study Selection

**Figure 1:**
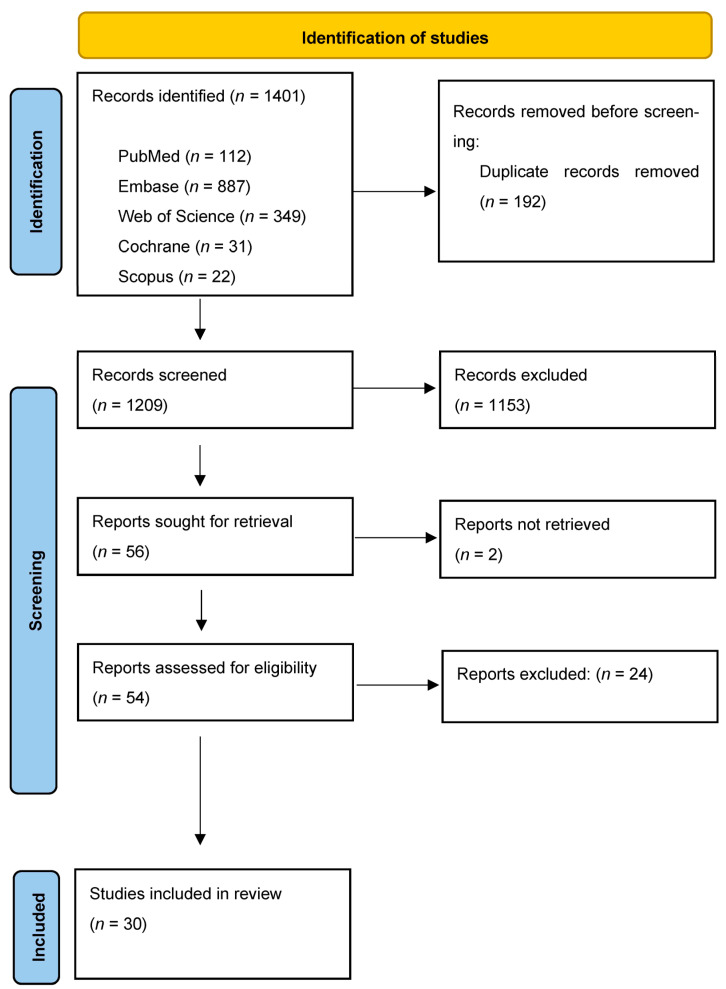
Study selection process for this systematic review.

The study selection process is summarized in Figure 1. The electronic search resulted in 1401 studies. After removing duplicates, 1209 articles remained for abstract screening. Of these, 54 articles were read in full, 24 were excluded. The remaining 30 studies were selected for inclusion.

In total, 30 studies met the selection criteria for inclusion in the review [8,11,12,13,15,16,21,22,23,24,25,26,27,28,29,30,31,32,33,34,35,36,37,38,39,40,41,42,43,44]. The main characteristic of each study is shown in Table 1. Selected articles were published between 2008 and 2023 and were a combination of comparative and observational studies. Of the 30 studies, 5 were single-arm observation studies [8,12,22,33,34]; 9 were comparative observations of the effects of diabetes [26], comparisons between teeth and implants [3,38], periodontitis history [15], osteopenia [39], smoking [41,42], insertion torque [30], and ridge regularization [43], while 17 comparative studies were interventional and randomized with respect to loading protocol [13,23,35], implant design (surface/type, abutment design) [16,24,25,27,28,41,42], surgical protocol (flap vs. flapless, osteotomy, CGF) [21,32,44], medication protocol [11], and post-operative protocol (PEMF, laser/photobiomodulation) [31,36,37].

#### Assessment and Risk of Bias

**Figure 2:**
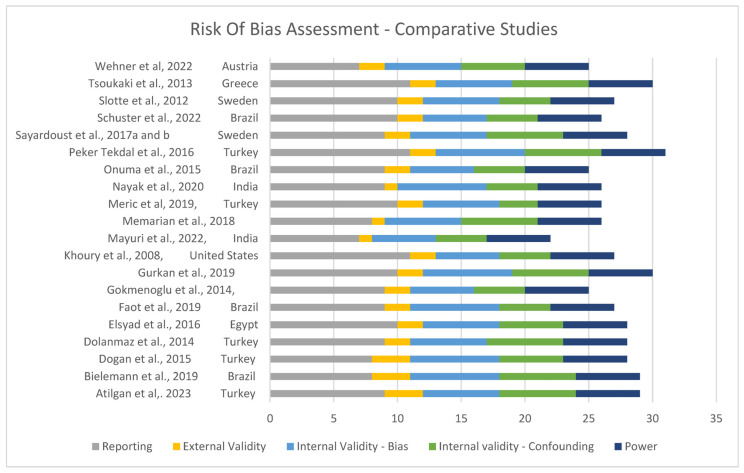
Risk of bias for comparative studies [11,13,14,16,21,23,26,27,28,30,31,32,35,36,37,39,40,41,42,43,44].

**Figure 3:**
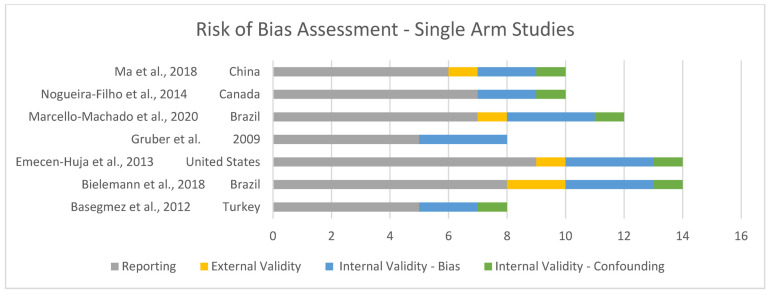
Risk of bias for single-arm studies [8,12,22,29,33,34,38].

Risk of bias was assessed and is shown in Figure 2 and Figure 3. Overall, the quality of the included studies was considered high, with most scoring GOOD (7 of 30) [16,21,31,33,35,38,39] or EXCELLENT (19 of 30) [8,11,13,15,23,26,27,28,29,30,32,34,36,37,40,41,42,43,44].

### 3.2. Study Characteristics

Table 1 summarizes the demographic characteristics of the study participants. In total, 710 patients received 1329 implants (an average of 1.87 implants per participant). The number of implants per study ranged from 11 [12] to 108 [35], and the sample size ranged from 10 patients [24] to 78 [33]. The follow-up period varied from post-surgical baseline [15,26] to 24 months [35], although the scope of this systematic review considered only data up to 12 weeks following implant placement. In nine studies, there was no mention in regards to the bone site or the location for implant placement [12,13,22,25,26,31,35,38,44]. Twelve studies focused on mandibular implant placement [8,21,23,24,27,28,29,30,34,36,39,43], and only one study focused on maxillary jaw rehabilitation [40]. Full mandibular overdenture rehabilitation was the focus of 8 studies [8,23,24,28,30,34,36,43]. Three studies used both immediate and conventional loading protocols [13,23,35], while conventional loading was examined in 13 studies [8,16,22,24,26,30,34,37,38,41,42,43,44], and immediate loading was examined in 3 studies [12,27,39]. Eleven studies did not provide any information regarding the loading protocol chosen [11,15,21,25,27,29,31,32,33,36,40].

A total of 32 biomarkers were analyzed. The biomarkers included in the review were (including number of studies for each biomarker): interleukin (IL)-1β (19 studies) [8,11,12,13,16,23,24,25,26,28,29,30,31,32,34,35,36,37,43]; Tumor Necrosis Factor-Alpha (TNF-α) (17 studies) [8,13,16,21,23,24,26,29,30,32,34,35,37,38,41,42,43]; IL-6 (10 studies) [8,16,23,25,29,32,34,38,41,42]; IL-10 (7 studies) [8,16,23,29,32,34,38]; IL-8 (6 studies) [16,25,29,38,41,42]; IL-12 (4 studies) [16,29,32,38]; IL-17 (3 studies) [15,16,21,32]; RANKL (4 studies) [16,21,33,40]; sRANKL (3 studies) [27,39,44]; Il-7 (2 studies) [21,29,32]; Interferon-gamma (INF-γ) (2 studies) [16,32]; IL-1α [16,21,38]; IL-2 (2 studies) [16,32]; IL-4 (3 studies) [16,32,38]; Chemokine Motif Ligand 3/Macrophage Inflammatory Protein 1-alpha (CCL3/MIP-1α) (3 studies) [16,25,32]; Eotaxin (2 studies) [29,32]; Chemokine Motif Ligand 2/Monocyte Chemoattractant Protein-1 (CCL2/MCP-1) (2 studies) [29,32]; Chemokine Motif Ligand 4/Macrophage Inflammatory Protein 1-beta (CCL4/MIP-1β) (2 studies) [29,32]; prostaglandin E2 (PGE2) (2 studies) [22,31]; and, in single studies, IL-1ra [32]; Granulocyte Colony-Stimulating Factor (G-CSF) [32]; Granulocyte-macrophage Colony-stimulating factor (GM-CSF) [32]; Interferon-Alpha (INF-α) [32]; IL-2R [32]; IL-5 [32]; IL-13 [32]; IL-15 [32]; Chemokine Motif Ligand 5/regulated on activation, normal t-cell expressed and secreted) (CCL5/RANTES) [32], and C-Reactive Protein (CRP) [16]. Also reported in six studies investigating RANKL and sRANKL was levels of Osteoprotegerin (OPG) [16,21,27,33,39,40]; in four studies, this was expressed as a RANKL:OPG ratio [21,33,39,40].

Antibiotics were given prophylactically in six studies as either Amoxicillin 2 g [11,16,28,32,42] or Amoxicillin 1000 mg + Clavulanic Acid 250 mg 5 days prior to surgery [26]. One study compared no antibiotic use with a protocol involving Amoxicillin 2 g pre-operatively and 500 mg three times per day for 7 days, post-operatively [11]. Post-operative antibiotics were given in 10 studies as either 500 mg Amoxicillin three times daily (8 studies) [8,11,23,27,30,37,38,43], clindamycin 300 mg plus 2 g phenoxymethylpenicillin twice daily [13], or clindamycin 900 mg twice daily [39]. No antibiotic use was reported in 13 studies [12,15,21,22,24,25,29,31,33,34,35,36,40]. Post-operative Chlorohexidine usage was described in 12 studies [11,13,27,28,29,37,38,39,40,41,42,44] at concentrations between 0.1% and 0.2%.

Treatment variables examined among the studies included immediate loading protocols [13,23,35], vertical location of the abutment/implant connection [24], the use of concentrated growth factors [21], modifications in the abutment material [16,25], implant surface modifications [27,42], the use of photobiomodulation [31], LED/laser photostimulation [36] or a pulsed electromagnetic field [37], the type of overdenture retention [28], osteotomy preparation techniques [32,40], insertion torque [30,32], antibiotic use [11], flap compared to flapless techniques [44], surgical time [40], and bony reduction at the time of surgery [43]. Other variables examined include a history of aggressive periodontitis [15], type 2 Insulin-Dependent Diabetes Mellitus [26], smoking [42] and osteopenia [39].

### 3.3. Individual Biomarker Outcomes

**Figure 4:**
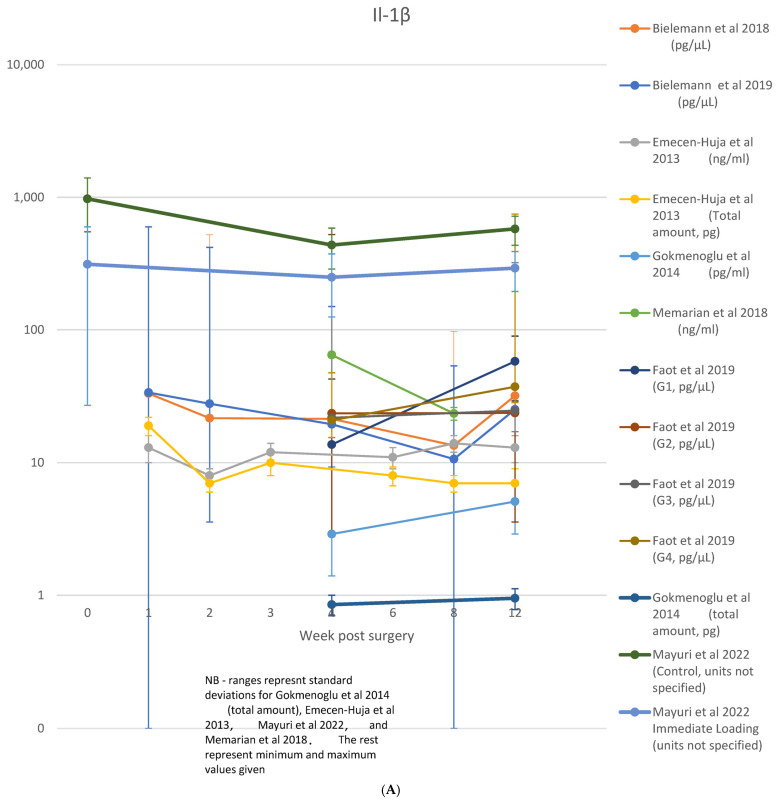

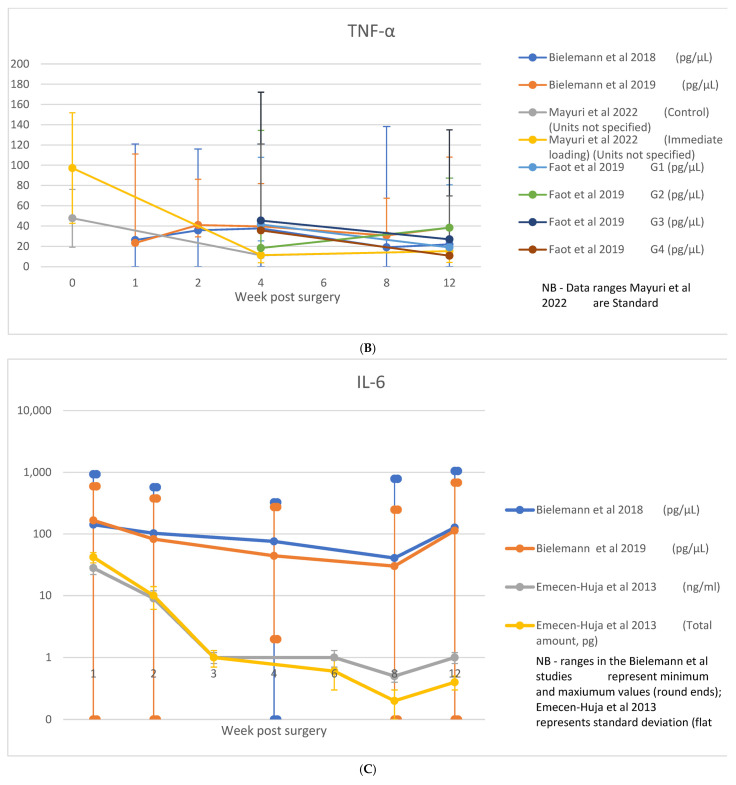

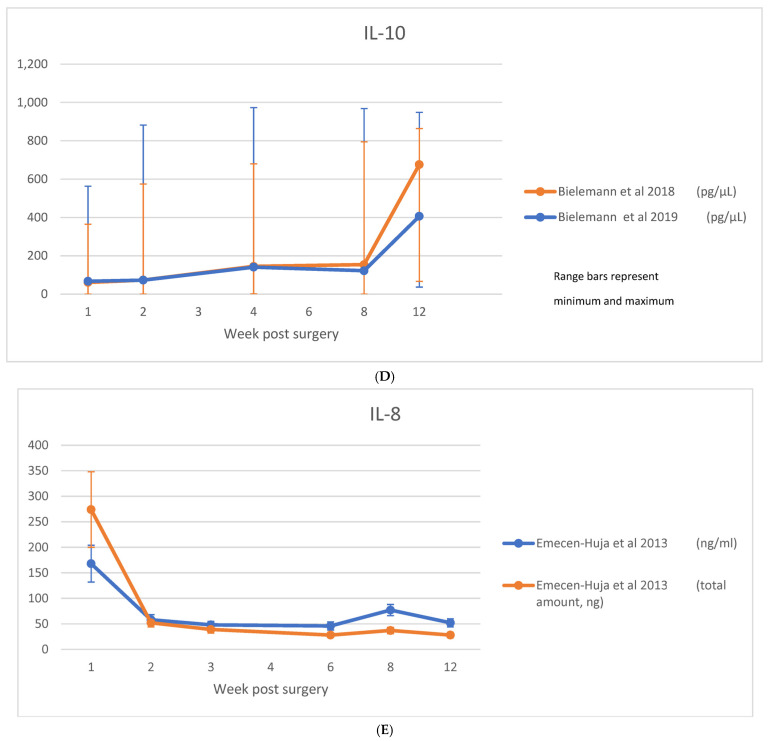

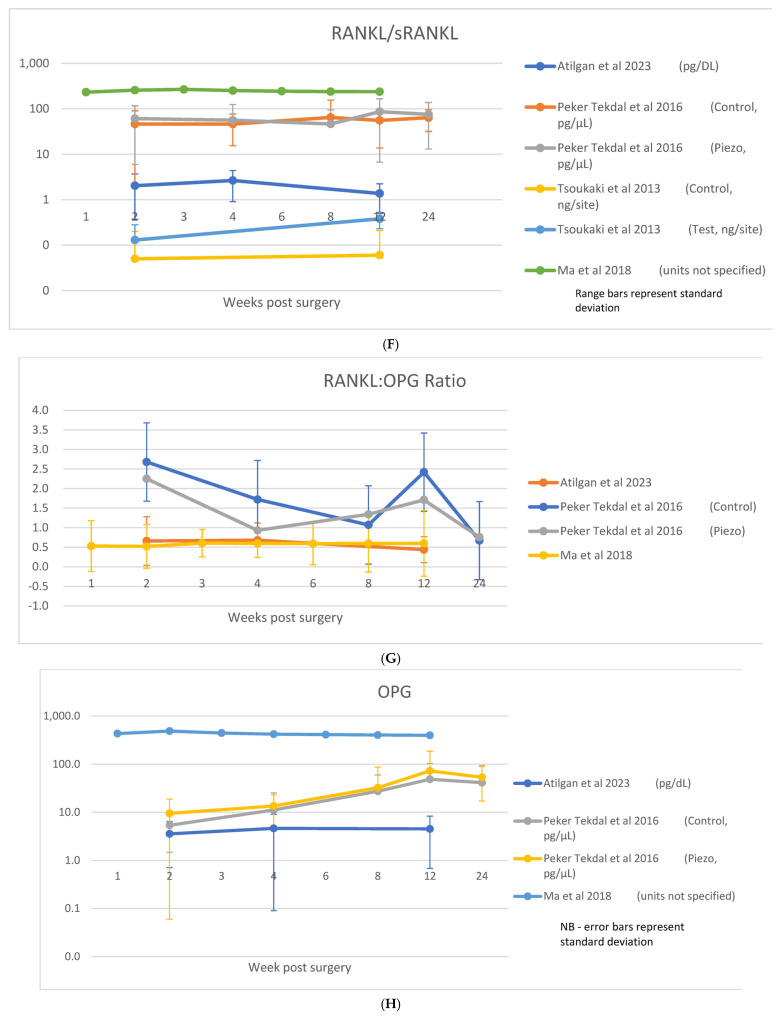
(**A**) IL-1β data; (**B**) TNF-α data; (**C**) IL-6 data; (**D**) IL-10 data; (**E**) IL-8 data; (**F**) RANKL/sRANKL data; (**G**) RANKL/OPG data; (**H**) OPG data [8,21,23,29,30,31,33,35,36,40,44].

A table showing analyzed biomarkers and respective timepoints across the included articles is attached in Appendix A. Raw data on cytokine levels (concentration or absolute amount) were variably reported. Among those studies where the biomarkers were considered, IL-1β was reported in nine studies [8,11,23,28,29,30,31,35,36], TNF-α in five studies [8,21,23,30,35], IL-6 in three studies [8,23,29], IL-8 in one study [29], IL-10 in two studies [8,23], sRANKL/RANKL in four studies [21,33,40,44], OPG in four studies [21,33,40], and RANKL:OPG ratio in three studies [21,33,40]. These data are shown in Figure 4A–H.

### 3.4. Interleukin 1-β (IL-1β)

IL-1β was the most extensively examined marker, featured in 19 studies [8,11,12,13,16,23,24,25,26,28,29,30,31,32,34,35,36,37,38] across timepoints from 1 [12] or 2 days [13,35] to 3 months [8,13,16,23,24,29,31,32,34,35,37,43]. Detectable levels were observed in all studies. A single timepoint within the osseointegration period was observed in three studies [11,25,28], while two timepoints over an unspecified range (>10 days) were analyzed by [12]. Of those studies, seven disclosed [8,23,29,32,36,37,43] a peak at the earliest observation point with a progressive reduction; while others followed up the decrease into the levels along 2 days [35], 1 week [23,29,43], 2 weeks [32,37], and 4 weeks [36]. This trend was also observed in three other studies [13,16,34]; however, the results were not statistically significant. In four studies, no significant changes were observed across the interleukin levels. [8,24,26,31]. A single study observed increased levels between 4 and 12 weeks [30], while a second peak in the interleukin levels was reported at week 12 [8,23,31]. As shown in Figure 4A, nine studies only presented the raw levels of the IL-1β identified across their quantitative analysis [8,11,23,28,29,30,31,35,36].

Variables examined not affecting IL-1β expression include antibiotics [11], piezoelectric osteotomy technique [32], photobiomodulation [31], insertion torque [30], healing abutment type [16], ridge reduction at time of surgery [43], pulsed electromagnetic field application [36], photodynamic stimulation [36], and diabetes [26]. Regarding protocols in prosthodontics, immediate loading revealed higher expression in overdenture cases [23], which was not seen across other prosthetic protocols [13]. Edentulism was positively correlated with lower overall levels of IL-1β [43]. Clinically, both smoking [8] and plaque levels [43] were correlated with elevated expression.

Healing outcomes such as probing depth, marginal bone loss, and RFA were not correlated with IL-1β levels in most of the studies analyzed [8,11,12,16,21,25,32,35,36,37]. Concerning marginal bone levels, no correlation with IL-1β was identified [23,26,28], although the level of IL-1β was disclosed in one study only, following an interval of 6 months [23]. Although no effect on probing depth was observed in seven studies [23,26,28,30,31,33,43], one survey reported PD tended to be higher in groups with greater expressions of the interleukin [24]. No effect on RFA was observed in seven studies [23,26,28,30,31,34,43], while the positive correlation with IL-1β expression was observed at 2 days post-surgery in a single study [13].

With respect to implant complications, one study [13] described complications in a broad sense, including bone dehiscence at the time of surgery, instability at the time of placement, and loss or loosening of the implant. A positive correlation with interleukin levels was identified at day 14, while a negative correlation was disclosed at day 28. In only one study [34] a negative correlation was seen between interleukin expression and implant failure throughout the observation period; however, this correlation was considered extremely weak [8] performed subgroup analysis between survival and loss groups and found no difference between IL-1β at any timepoint.

### 3.5. TNF-α

TNF-α was examined in 16 studies, across timepoints ranging from 1 day [42] to 3 months [13,16,38,42,43]. Two studies [16,32] found levels below detectable limits, whilst four studies observed no significant or consistent trend across timepoints [21,29,30,38]. Stable levels were observed in the control groups of two studies, one comparing the use of CGF [21] and another where immediate loading protocol was adopted [23]. A trend for an early peak and progressive reduction was observed across seven studies [8,13,21,35,37,42,43], with this peak occurring between 2 days [13,35], 7 days [21,42,43], and 2–4 weeks [8,21,37]. A gradual increase without reduction through the integration phase was observed in two studies involving removable overdenture prostheses [23,34]; however, in one study, this trend was reported only in the immediate loading group [23]. Raw data on TNF-α were reported in five studies [8,21,23,30,35] and are shown in Figure 4B.

Healing outcomes were not analyzed with respect to TNF-α levels in seven studies [8,21,24,32,35,37,38]. No correlation with marginal bone levels was reported in two studies [26,34], while one study reported a weak negative correlation between levels and MBL at week 4 in the high insertion torque group (>45 nCm) [30]. Three studies reported no correlation with increased probing depths [26,30,34], while a single study found a negative correlation [43]. RFA values saw conflicting observations with respect to TNF-α levels, as two studies reported no correlation [26,34]. In comparison, although four studies reported negative correlations [23,30,37,43], in two of those, a positive correlation was seen at a single timepoint (4 weeks) [23] and along a specific insertion torque subgroup [30]. Positive correlations with RFA were reported in two studies and involved a smoking subgroup [42] or RFA at baseline and TNF-α levels at 90 days [13]. Higher TNF-α levels were positively correlated with Visual Analogue Scale (VAS) scores across time [42].

Four studies reported a correlation with failure rates [8,13,34,42], whereas three identified a negative correlation with TNF-α levels [13,34,42]. The correlation was observed as early as day 1 [42] and also between days 2 and 14. [13]. Sayardoust et al. [42] reported levels in the failure group to be 14x lower at day 1; however, an opposite trend was observed when the failure was analyzed in smokers, where TNF-α levels were elevated three-fold compared to the survival group.

Several variables were observed to affect TNF-α expression. Immediate loading was examined in four studies [13,23,37,43], with two of those reporting an elevation in TNF-α levels [23,37]. Bone atrophy in edentulous jaws revealed a delay of 2 weeks in the peak of TNF-α expression when compared to non-atrophic patients [8]. Time since edentulism was also associated with lower overall levels [43], while type 2 bone was associated with elevated levels [34]. Alveolar reduction at the time of surgery resulted in a prolonged elevation in levels, taking longer to return to baseline than controls [43]. Application of CGF at the time of surgery was associated with elevated levels at weeks 2 and 4 [21]. Smoking was reported in two studies and saw conflicting effects. One study [42] reported no difference in TNF-α levels between smokers and non-smokers overall, except when specifically examining softer bone in smokers where levels were found to be lower. The second [8] observed overall lower levels and an earlier peak in TNF-α levels (2 vs. 4 weeks) among smokers compared to non-smokers. Variation of the abutment type [13,16] and insertion torque [8,30] had little effect on TNF-α levels.

### 3.6. IL-6

IL-6 was examined in nine [8,16,23,25,29,32,34,38,41,42] studies across timepoints ranging from 1 day [42] to 3 months [8,16,23,29,32,34,41,42]. Detectable levels were found in all studies, with a single timepoint observed in one study [25] and two finding no trend over the observation periods [34,38]. A consistent trend for progressive reduction over time was observed in the six remaining studies [8,16,23,29,32,42], with peaks occurring in the earliest observation points of each study following surgery (ranging from 1 day [42], 1 week [8,16,23,29], and 2 weeks [32]). A second peak at 12 weeks was observed in two studies [8,23], both involving overdenture prostheses and coinciding with the time at which final prostheses were attached. Raw data were reported on IL-6 in three studies [8,23,29], which are shown in Figure 4C.

Among these studies, four reported a correlation with healing parameters [8,23,34,42]. Few significant correlations were seen with some notable exceptions. Marginal bone loss was strongly correlated at 90 days with IL-6 levels and positively correlated with pain scores at 7 days in one study [42]. IL-6 expression was 3–4 times higher in the failure group at 1 and 7 days [42]; however, this was not seen in two studies [8,34]. A modest correlation with ISQ values was seen at week 6 in one study [23], but not in two others [34,42]. Probing depth saw no correlation to IL-6 levels [8,23,34].

Abutment type [16,25] or the use of piezoelectric surgery for osteotomy preparation [32] had no effect on IL-6 expression. Smoking produced conflicting results; increased levels were observed in two studies [34,42] but only in the later phases of healing [4–12 weeks] in one study [42], whilst a negative correlation at week 4 was found in another study, with a general non-significant trend for lower levels among smokers [8]. Immediate loading was associated with lower levels in overdenture cases, likely relating to tissue trauma from loaded prostheses [23].

### 3.7. IL-10

IL-10 was examined in seven studies [8,16,23,29,32,34,38] across timepoints ranging from 1 week [8,16,23,29] to 3 months [8,16,23,29,32,34,38]. Levels were below detection in two studies [16,32], and a further two observed no consistent trend over the observation period [29,38]. Three studies [8,23,34] observed a progressive increase over the 3 months encompassing osseointegration. Raw data were reported for Il-10 in two studies [8,23], which are shown in Figure 4D.

Implant failure was correlated with higher levels at weeks 2, 4, and 8 compared to those that were successfully integrated in one study [8]. No correlation with ISQ values was seen [34], and one study described a negative correlation with probing depth [34].

Correlation with smoking was reported in two studies, with both a positive [34] and strong negative [8] association found. With respect to other variables, immediate loading was the only randomized variable examined, which saw effects limited to an elevation in IL-10 levels in the immediate loading group at 12 weeks compared to delayed loading [23]. A correlation with other biomarkers was described in one study [23], with a strong positive correlation between IL-10 and both TNF-α and IL-1β at week 8 and a high negative correlation between IL-6 at week 2.

### 3.8. IL-8

IL-8 was examined in seven studies [11,16,25,29,32,38,42] across timepoints ranging from 1 day [42] to 3 months [16,29,32,38,42] and was detected in all studies. A single timepoint was observed in two studies [11,25]. One study found no temporal trend [38] and a non-significant trend for reduction was observed in another [16]. Two studies [29,42] observed an early peak at 1 day, followed by a reduction to low levels through the observation period. Raw data were reported for IL-8 in one study [29], which are shown in Figure 4E.

A single study reported failed implants to have levels of IL-8 elevated 2–3 times higher than the survival group at 7 and 14 days, along with a positive correlation with pain and IL-8 during the first week in the failure group [42].

Little effect of treatment variables was observed across studies, including with respect to abutment type [16,25], the use of piezoelectric surgery [32], antibiotics [11], smoking [42] and implant surface [42]. Limited correlation was observed with respect to clinical outcomes, including the effect on bone levels and ISQ [42].

### 3.9. IL-12

IL-12 (including IL-12A or IL-12p70) was examined in four studies [16,29,32,38] at timepoints ranging from 1 week [16,29] to 3 months [16,29,32]. It was below detectable levels in one study [16] and showed no consistent trend across time or with respect to clinical variables in two others [29,38]. A time-dependent decrease in levels was observed in a single study, with a peak occurring at 2 weeks following implant placement (the earliest timepoint in this study) [32].

### 3.10. IL-17A, IL-17E

IL-17A was examined in three studies [15,16,32] at timepoints ranging from 1 week [16] through to 3 months [16,32], whilst IL-17E (IL-25) was examined in a single study at timepoints 10 days and 4 weeks [15]. In two studies, IL-17A was found to be below detectable limits [16] or was detectable but showed no consistent trend across timepoints [32]. The remaining study [15] examined IL-17A and IL-17E expression in the first 4 weeks of osseointegration among patients with a history of treated aggressive periodontitis and periodontally healthy controls. Increased IL-17A expression was observed in patients with a history of aggressive periodontal disease; an increase of IL-17A from day 10 to 4 weeks was also observed in this group but not in periodontally healthy controls. IL-17E levels increased from day 10 to week 4 and were lower in patients with a history of aggressive periodontal disease. IL-17A and the ratio of IL-17A:IL-17E were correlated with increased marginal bone loss at 4 weeks; however, this effect was not observed at 6 months.

### 3.11. RANKL/sRANKL and OPG

RANKL was examined in four studies [16,21,33,40], with a further three examining sRANKL [27,39,44] across timepoints ranging from 1 week [16,33,39,44] to 3 months [16,21,27,33,40,44].

In addition, six studies [16,21,27,33,39,40] also examined the decoy receptor of RANKL, Osteoprotegerin (OPG), at timepoints ranging from 1 week [16,33,39] to 3 months [16,21,27,33,40]. Four studies also provided analysis with respect to the ratio of RANKL:OPG [21,33,39,40]. Raw data were reported for sRANKL/RANKL in four studies [21,33,40,44], OPG in four studies [21,33,40] and RANKL:OPG ratio in three studies [21,33,40], which are shown in Figure 4F–H.

Changes in RANKL/sRANKL levels over time were inconsistent among studies. Four studies observed an early peak in levels between 2 [40], 3 [33] or 4 [21,39] weeks, which then reduced over time (in one of these studies [40], this was seen in absolute values only, with the concentration of RANKL being unaffected). One study [16] reported an increase in RANKL/sRANKL levels over time; this was also seen in the bone level subgroup of a second study with no changes observed in the tissue level controls [27]. In addition to the study reporting no change over time among the tissue-level implants [27], no changes across timepoints were reported in another study [44].

OPG was generally observed to increase across the observation period [16,21,33,39,40]. three of these observed highest concentrations at 12–16 weeks [16,39,40]; in three studies, an early peak was observed at 2 [33] or 4 [21,27] weeks.

The ratio of RANKL:OPG was generally observed to decrease as a function of decreasing RANKL levels and increasing OPG levels. A progressive reduction was reported in two studies [39,40], as well as in the test group of a second study [21]; a trend for reduction over time was seen in the control group of this study but did not reach significance. One study reported no clear trend [33].

Three studies did not analyze RANKL expression with respect to clinical outcomes [21,39,40]. One study observed a negative correlation between ISQ values and both RANKL and OPG [33]. A strong negative correlation was reported between both GI and PI and OPG in one study [27]. In one study comparing flap vs. flapless placement protocols, higher levels of marginal bone loss were reported in the flap group, but this was not correlated with changes in sRANKL expression [44].

Osteopenia had no effect on levels of sRANKL and OPG [39], and there was no significant effect seen comparing piezoelectric osteotomy sites to conventional rotary instrumentation, except with respect to RANKL total levels which was lower in the piezo group [40]. Higher levels of both RANKL and OPG were observed in the implants receiving CGF compared to controls [21], but there was no change in the RANKL:OPG ratio. Bone-level implant placement also appeared to reduce the concentration of both OPG and sRANKL compared to tissue-level placement; this was not observed when total levels were measured [27].

### 3.12. Chemokines

Chemokines were only examined in a small number of studies. CCL3/MIP1-a was examined in four studies [16,25,29,32] across timepoints ranging from 1 week [16,25,29] to 3 months [16,29,32]. It was detectable in all studies, with two noting a time-dependent decrease that reached significance by weeks 3–4 [29,32]. No change in levels was observed in another [16], whilst a single timepoint was observed in the final study [25].

The chemokines Eotaxin (CCL-11), MCP-1 (CCL-2), and MIP-1B (CCL-4) were examined in two studies [29,32] across timepoints ranging from 1 week [29] to 3 months [29,32]. Eotaxin was found to be below detectable levels [32] or showing no significant trend [29]. MIP-1B (CCL-4) was observed to follow an early peak in the first 2 weeks before sharply decreasing by weeks 3–4. MCP-1 (CCL-2) followed a time-dependent decrease from the second week [32] and was below detectable limits in the other study [29].

Prostaglandin E2 (PGE2) was examined in three studies [22,31,36] at timepoints from 4 to 12 weeks. One study was a single timepoint at 3 months [22] and observed a correlation between levels and both gingival index and probing depths. No correlation with probing depths was observed in the other study that reported this [31]. A progressive reduction in expression between 4 and 8 weeks was reported by Memarian et al. [36].

RANTES (CCL-5) was examined in a single study across timepoints from 2 weeks to 3 months [32]. No consistent trend in levels was observed over time, but a significantly lower overall level was observed in the piezo-osteotomy group compared to conventional drill preparation.

### 3.13. Other Biomarkers

Several cytokines were examined in a small number of studies with no consistent trend: IL-7 (two studies) [29,32], IL-4 (three studies) [16,32,38] and a single study [32] for the biomarkers IL-1ra, GM-CSF, IL-5, IL-15.

IL-1a was examined in two studies [16,38]. Both observed a trend for reduction over the first 3 months of healing but neither reached statistical significance.

A single study [32] examined GCSF, INF-a, INF-y, IL-2, IL-2r, and IL-13, with the general trend for a peak at 2 weeks with a progressive reduction through to week 8 or 12. One additional study [16] also examined INF-y and IL-2 but found them below detectable limits. These cytokines were not analyzed with respect to MBL or Probing depths.

## 4. Discussion

A consistent trend for an early peak followed by a reduction in pro-inflammatory biomarkers is observed across the studies included in the systematic review. For many studies and biomarkers, this was observed at the earliest observation timepoint following placement. This effect was observed for seven studies examining IL-1β [8,23,29,32,36,37,43], seven studies examining TNF-α [8,13,21,35,37,42,43], six examining IL-6 [8,16,23,29,32,42,45], three studies examining IL-8 [16,29,42], and Il-12 [32]. Histological studies examining the healing timeframes of transmucosal dental implants in humans [46,47] have observed the formation of a functional epithelial barrier between the three- and four-week mark, which undergoes maturation up to 8 weeks. A reduction in the density of inflammatory cells occurs from weeks 4 to 8. Regarding hard tissue, the apposition of new bone to the implant surface appears no earlier than two weeks [48]. These observations are consistent with the peak in pro-inflammatory markers generally observed in the systematic review.

Delineating temporal changes and the exact timing of peak inflammation can be difficult when various combinations of timepoints are used across studies. The most useful data, in the context of our investigation, are those studies that observe multiple timepoints within the osseointegration period. Several studies, although observing across the osseointegration phase, only observed one [11,22,24,25,28,39] or two [12,15,16,26,27,30,32,36,38] timepoints within the first 12 weeks. While these data show the presence and fluctuation of biomarkers, extrapolating an approximate peak in these markers is not possible based on the timepoints provided. This is a caveat with which trends need to be interpreted.

A second peak in IL-1β [8,23,31,34], TNF-α [23,34], and IL-6 [8,23] was observed around 12 weeks. In these studies, these timepoints either coincided with the delivery of a final abutment or the use of overdentures, suggesting elevation relating to tissue trauma.

Failure to achieve osseointegration (early failure) was analyzed with respect to IL-1β, TNF-α, IL-6, IL-8, and IL-10. Elevated IL-6 [42], IL-8 [42] in the first two weeks, and elevated IL-10 throughout weeks 2, 4, and 8 [8] were all positively correlated with early failure, while a negative correlation between early failure and TNF-α between 1 and 14 days was observed [13,34,42]. No such relation was observed for IL-1β [8,13,43]. A dysregulated inflammatory response may be related to early implant failure; however, further data are needed to draw mechanistic conclusions.

In the present systematic review, the direct correlation between biomarker expression and clinical outcomes was inconsistently reported, limiting our ability to comment with a high degree of certainty on the effect of these cytokines directly on clinical outcomes. Most studies examined primarily investigated clinical parameters as an outcome of treatment variables, while separately examining the influence of treatment variables on biomarker expression to measure healing on a molecular level. Some interesting correlations were reported, however. A positive correlation between marginal bone loss was reported with IL-6 [42] and IL-17A [15], while a negative correlation was observed with TNF-α [30]. Early marginal bone loss may play a predictive role in the development of peri-implant disease. A retrospective study by Galindo-Moreno et al. [49] observed implants with a marginal bone loss of 0.48 mm or greater by 6 months after loading and invariably saw the progression of bone loss beyond 2 mm by 18 months. Based on a limited sample size in the current review, it is difficult to account for the effect of clinical variables in the placement and draw conclusions. The available evidence suggests that an over- or underexpression of certain biomarkers could have an effect on early marginal bone levels, which may in turn have an effect on the incidence of peri-implant disease over time. Whether this is a causal relation based on shared immunology (that is, early marginal bone loss indicated a strong inflammatory phenotype, which determines the bone loss seen in the inflammatory response to subsequent bacterial challenges) or simply co-incidental (early bone loss due to placement variable creates and unfavorable anatomical position which influences bone stability) is unclear.

In the same study reporting IL-17A, an elevated level of IL-17A was also observed in the patient group with a history of aggressive periodontitis during osseointegration. A history of periodontitis [50], especially aggressive or severe forms [51], is strongly associated with an elevated risk of peri-implantitis. The observation that patients with a known overt pro-inflammatory response to a bacterial challenge (a history of aggressive periodontitis) also exhibit a stronger pro-inflammatory response to a separate and mechanistically distinct trauma (implant placement) is interesting in the context of the current investigation.

From the results of the current systematic review, it is clear there exists a significant overlap between those markers detected during the peri-implant disease process and those expressed during the first 12 weeks of osseointegration. Future research may be targeted at the longitudinal observation of a cohort of patients, specifically looking at markers identified in both peri-implantitis and the early stages of osseointegration. Those inflammatory markers identified in common include IL-1B, TNF-α, IL-6, IL-17, IL-10, IL-4, IL-8, and RANKL [52] Whether variations in the expression of certain biomarkers can be correlated with the future incidence of peri-implant diseases may allow the delineation of a peri-implantitis susceptible patient based on inflammatory profile expressed in the early stages of healing. Ideally, this would allow a clinician to tailor recall periods for preventative care based on individual, not collective risk, and time interventions at a more effective timepoint.

As a limitation of this review, very few studies provided raw data surrounding the total amount or concentration of the investigated markers and simply reported on averages/ranges or changes across timepoints. The aim of our review was to identify markers present but also to observe whether there were significant variations among individuals. There are limited data from which to draw meaningful conclusions; however, the stated ranges were generally quite large. The implication of large detectable ranges among a single study cohort suggests, as one possibility, that there is a significant variation in expression among individuals. Whether this bears any clinical or prognostic relevance is unclear at the current time and requires additional longitudinal study.

## 5. Conclusions

In conclusion, the current evidence suggests that either overexpression or underexpression of specific biomarkers may impact early marginal bone levels. To further understand this relationship, additional well-designed longitudinal studies with larger sample sizes and control groups are needed, encompassing a broader range of cytokines. Additionally, longitudinal studies should be conducted to confirm whether early expression of pro-inflammatory cytokines or underexpression of anti-inflammatory cytokines contributes to bone loss around implants over time.

## Figures and Tables

**Table 1:** Summary characteristics of included studies.

Author, Year, Country, Study Design	*N*	Implants (*n*), Brand	Follow-Up	Outcome Variables	Treatment Variables	Bone Site/Prosthetic Treatment	Abutment or Restoration	Biomarkers	Antibiotics	Methodology for Analysis
Atilgan et al., 2023 [21], Turkey (RCT)	20	40, T6 NucleOSS	2, 4, 12 weeks	RFA, MBL, PI, GI, PD, GBI	Concentrated Growth Factors	Mandibular Bilateral Edentulous Spaces	Abutment Only	TNF-α, RANKL, OPG	No	
Basegmez et al., 2012 [22], Turkey (Long)	28	72, Branemark/Nobel Biocare	3, 6, 12, 18 months	PI, GI, PD		Totally Edentulous (Jaw Not Specified)	3 Months, Not Specified	PGE2	Not Specified	Assay Designs, Inc., Ann Arbor, MI, USA
Bielemann et al., 2018 [8], Brazil (Long)	30	60, Neodent Brazil	1, 2, 4, 8, 12 weeks	PI Calculus, PD, BOP, GI		Mandibular Overdenture	3 Months	IL-1B, IL-6, IL-10, TNF-a	Amoxil 500 mg TID 7 Days	ELISA (Duoset Kit, R&D, Minneapolis, MN, USA)
Bielemann et al., 2019 [23], Brazil (RCT)	20	40, Neodent Brazil	1, 2, 4, 8, 12 weeks	ISQ, PI, GI, Calculus, PD, BOP	Immediate vs. Delayed Loading	Mandibular Overdenture	Immediate (Test), Delayed (Control)	IL-1B, IL-6, IL-10, TNF-a	Amoxil 500 mg TID 7 Days	ELISA (Duoset Kit, R&D, Minneapolis, MN, USA)
Boynuegri et al., 2012 [24], Turkey (RCT)	10	40, Straumann, Switzerland	3, 6, 12 months	PD, GI, BOP, PI	Location of Abutment Interface	Mandibular Overdenture (Dolder Bar)	3 Months (Dolder Bar)	IL-1B, TNF-a	No	ELISA (Biosource, Minneapolis, MN, USA)
De Wilde et al., 2015 [25], Belgium (RCT)	12	13	1 week	-	Hydroxyapatite vs. Commercially Pure Ti	Partially Edentulous	NA	IL-6, CCL-3, IL-8, IL-1B	No	mRNA (miRNeasy Mini Kit, Qiagen, Venlo, The Netherlands)
Dogan et al., 2015 [26], Turkey (Comparative Obs.)	20	39, Straumann SLActive, Switzerland	Baseline, 1, 4, 7 months	PI, GI, PD, BOP, CAL, KGW, ISQ, MBL	Diabetic vs. Non-Diabetic	Partially Edentulous	Restored at 4 Months	IL-1B, TNF-a	Amoxil 1000 mg + Clavulanic Acid BID 5 days Prophylactic	ELISA Conc. and Total amount (Orgenium Laboratories Oy/Ani Biotech Oy, Minneapolis, MN, USA)
Dolanmaz et al. 2015 [27], Turkey (RCT)	47	47, Straumann AND NucleOSS	1, 3 months	PI, GI, PD, RFA	Modification of Implant Surface (SLActive)	Mandible Sites	Abutment Only	sRANKL, OPG	Amoxil 500 mg TID 7 Days	ELISA (Biovendor, Minneapolis, MN, USA)
Elsyad et al., 2016 [28], Egypt (RCT)	32	64, Dentarum, Germany	2 weeks, 6 months	Plaque, Bleeding, PD, RFA, HBL, VBL	Locators vs. Magnetic Attachment	Mandibular Overdenture	Immediate Restoration	IL-1B	Amoxil 2 g (Prophylactic)	Sandwich ELISA (FIRICH ENTERPRISES CO, LTD, New Taipei City, Taiwan)
Emecen-Huja et al., 2013 [29], United States (Comparative Obs.)	40	40, Astra; Straumann; Zimmer	1, 2, 3, 6, 8, 12 weeks	PI, GI	Comparison to Adjacent Tooth and Nonadjacent Teeth	Mandibular Single Tooth	Abutment Only	IL-1b, IL-1ra, IL6, IL-7, IL-8, IL-10, IL-12, Eotaxin, MCP-1, TNF-a, MIP-1b (CCL-4)	No	Multiplex Bead Assay (Bioplex, Bio-Rad Laboratories, Berkeley, CA, USA)
Faot et al., 2019 [30], Brazil (Comparative Int.)	31	62, Neodent, Brazil	4, 12 weeks	GI, PIH, PI, Calculus, PD, BOP, RFA	Insertion Torque	Mandibular Overdenture	3 Months	IL-1B, TNF-a	Amoxil 500 mg TID 7 Days	Not Specified
Gokmenoglu et al., 2014 [31], Turkey (RCT)	15	22, Dentsply, Germany	4, 12 weeks	PI, GI, PG, BOP, RFA	Photobiomodulation	Single Tooth; Type 2 or 3 bone	Abutment Only	IL-1B, PGE2	No	ELISA (Dbendermed Systems, Vienna, Austria) Conc. and Total Amount
Gruber et al., 2010 [12], Austria (Long)	11	11, Nobelreplace Nobel Biocare, Sweden	1–10 days, >10 days	-		Immediate Placement and Loading	Immediate Restoration	IL-1B	No	ELISA (R&D Systems, Minneapolis, MN, USA)
Gurkan et al., 2019 [32], Turkey (RCT)	14	38, Biodenta Switzerland	2, 4, 8, 12, 24 weeks	Insertion torque, early healing index, PI, mGI, mPI, MBL	Piezosurgery vs. Conventional	Bilateral Posterior Maxilla	Abutment Only	GCSF, GM-CSF, INFa, INFy, IL-1B, IL-1RA, IL-2, IL-2R, IL-4, IL-5, IL-6, IL-7, IL-8, IL-10, IL-12, IL-13, IL-15, IL17, TNF-a, MIP-1a (CCL3), MIP-1b (CCL4), MCP-1 (CCL2), RANTES (CCL5), MIG (CXCL-9), IP-10, (CXCL10), Eotaxin (CCL-11)	Amoxil 2 g (Prophylactic)	Multiplex Bead Assay (Novex/xPONENT, ThermoFisher Scientific, Waltham, MA, USA)
Khoury et al., 2008 [11], United States (RCT)	20	20, Astra, Sweden; Zimmer, USA	1 week	PI, GI	Antibiotic (Prophylactic and Post-operative)	Bound Edentulous Site	Abutment Only	IL-1B, IL-8	Amoxil 2 g (Prophylactic), 500 mg Amoxil TID 7 Days (Test); None for control	ELISA (R&D Systems, Minneapolis, MN, USA)
Ma and Wu, 2018 [33], China (Retro)	78	78, Straumann, Switzerland	1, 2, 4, 6, 8, 12 weeks	Survival; complications; RFA, mPI, mSBI		Single Tooth Maxilla or Mandible	Abutment Only	RANKL, OPG,	No	
Marcello-Machado et al., 2020 [34], Brazil (Long)	16	32, Neodent Brazil	4, 8, 12, 24, 48 weeks	Bone density, PI, GI, calculus, PD, BOP, RFA, MBL		Mandibular Overdenture	Restored at 12 Weeks	TNF-a, IL-1B, IL-6, IL-10	No	ELISA (Duoset Kit, R&D, Minneapolis, MN, USA)
Mayuri et al., 2022 [35], India (RCT)	44	108, Nobel Biocare Sweden	2 days, 1 week, 4 weeks, 3, 6, 12, 24 months	PD, BOP, MBL, coronal height of tissue	Immediate vs. Delayed Loading	Partial Edentulism (Multiple Units)	Restored at 3 Days (Test); 3 Months (Control)	IL-1B, TNF-a	Not Specified	Quantitative PCR (BioRad, Berkeley, CA, USA)
Memarian et al., 2018 [36] (RCT)	12	36 Dio, Korea	4 weeks, 8 weeks	Periotest stability	LED/Laser Photostimulation	Mandibular, Overdenture (Not Clear)	Not Clear	IL-1B, PGE2	Not Specified	ELISA (Thermofisher, Waltham, MA, USA)
Meric et al., 2019 [15], Turkey (Comparative Obs.)	20	20, ISY Camlog Switzerland	Baseline, 4 weeks	MBL	Aggressive Periodontitis	Single Tooth Maxilla or Mandible	Abutment Only	IL-17A, IL-17E	No	ELISA (PeproTech, Cranbury, NJ, USA)
Nayak et al., 2020 [37], India (RCT)	19	40, ADIN Israel	2, 4, 6 weeks, 3, 6, 12 months	RFA, MBL	Pulsed Electromagnetic Field	Varied; Bone Type Specified	Restored at 3 Months	IL-1B, TNF-a,	1 g Amoxil Pre-Op; 500 mg TID post	ELISA (Thermofisher, Waltham, MA, USA)
Nogueira-Filho et al., 2014 [38], Canada (Comparative Obs.)	21	27, Ankylos, USA	1, 2, 4, 6, 12 months	PI, BOP, PD, CAL, RBL		Varied	Restored at 4 Months	IL-1a, Il-4, Il-6, Il-8, Il-10, IL-12p70, TNF-a, INFy	Amoxil 500 mg TID 5 Days	Quantibody Custom Array (Peachtree Corners, GA, USA)
Onuma et al., 2015 [39], Brazil (Comparative Obs.)	22	88, Titanium Fix, Brazill	1 week, 4 months	PI, GI, BOP, Suppuration, CAL, MBL	Osteopaenia	Mandible, Full, or Partial Fixed Bridges	Immediate Restoration	sRANKL, OPG	Clindamycin 900 mg BID 7 Days	Elisa (BioMedika, Vienna, Austria)
Peker Tekdal et al., 2015 [40], Turkey (RCT)	15	40 (38; 2 lost), Biodenta, Switzerland	2, 4, 8, 12, 24 weeks	MGI, MPI, PD (12 and 24 weeks), MBL, VAS, EHI, Surgical Time	Piezosurgery vs. Conventional	Bilateral Posterior Maxilla	Not Clear	RANKL, OPG	No	ELISA (Biovendor, R and D, Brno, Czech Republic)
Sayardoust et al., 2017a and b [41,42], Sweden (RCT and Comparative Obs.)	32	96, Branemark, Sweden; Nobel Biocare, Sweden; BioHelix, Sweden	1, 7, 14, 28, 60, 90 days	RFA, VAS	Smoking AND Implant Surface	Maxilla or Mandible	Restored at 90 Days	IL-6, IL-8, TNF-a	2 g Amoxil Prophylactic	Quantitative PCR (BioRad, Berkeley, CA, USA)
Schuster et al., 2022 [43], Brazil (Comparative Int.)	21	42, Neodent Brazil	7, 15, 30, 60, 90, 180 days	PI, Calculus, PD, BOP, GI, RFA	Ridge Regularization at Time of Surgery	Mandibular 2 Implant Overdenture	Restored at 3 Months	IL-1B, TNF-a	Amoxil 500 mg TID 7 Days	ELISA (Duoset Kit, R&D, Minneapolis, MN, USA)
Slotte et al., 2012 [13], Sweden (RCT)	18	54, Branemark/ Nobel, Sweden	2, 14, 28, 90 days	Bone Quality, Wound Healing Index, BOP, Sulcular Bleeding, RFA, Complications	Immediate vs. Delayed Loading	Partially Edentulous	Restored at 2 Days (Test); 3 Months (Control)	IL-1B, TNF-a	Clindamycin 300 mg + 2 g Phenoxymethylpenicillin BID 5 Days	Quantitative PCR (BioRad, Berkeley, CA, USA)
Tsoukaki et al., 2013 [44], Greece (RCT)	20	30, Osseospeed, ASTRA Sweden	1, 2, 6, 12 weeks	PI, GI, PD, MBL, Bacterial profiling, VAS	Flap vs. Flapless	Not Specified	Restored at 3 Months	sRANKL	Amoxil 1 g TID 4 Days	Elisa (BioMedika, Vienna, Austria))
Wehner et al., 2023 [16], Austria (RCT)	22	30, MIS Israel	1 week, 3, 6 months	MBL	Custom Milled Ti Abutments	Molar Sites (Jaw Not Specified)	Restored at 3 Months	CRP, INFy, TNF-a, IL-1a, IL-1B, IL-2, Il-4, IL6, IL-8, IL-10, IL-12A, IL-17A, Osteoprotegrin, RANKL, MIP-1a (CCL-3)	2 g Amoxil Prophylactic	ELISA (RayBitoech, Peachtree Corners, GA, USA)

Abbreviations: RCT—randomized control trial; Long—longitudinal study; Int—interventional; Obs—observational; RFA—resonance frequency analysis; MBL—marginal bone loss; PI—plaque index; GI—gingival index; PD—pocket depth; GBI—gingival bleeding index; BOP—bleeding on probing; ISQ—implant stability quotient; KGW—keratinized gingival width; HBL—horizontal bone loss; VBL—vertical bone loss; BID—two times a day; TID—three times a day; PIH—peri-implant health; mGI—modified gingival index; mPI—modified plaque index; mSBI; modified sulcular bleeding index; CAL—clinical attachment level quality a; ELISA- Enzyme Linked Immunosorbent Assay; IL—Interleukin; TNF—Tumor necrosis factor.

## Data Availability

The data generated in this research project are available for access by contacting the last author of this paper via email. They are stored electronically as Excel worksheets.

## Appendix A. Biomarkers and Their Respective Timepoints Across Studies

**Biomarker**	**Baseline—1 Day**	**2 Days**	**1 Week/** **7 Days**	**2 Weeks/15 Days**	**3 Weeks**	**4 Weeks/28–30 Days/1 Month**	**6 Weeks**	**8 Weeks/2 Months/60 Days**	**90 Days/12 Weeks/3 Months**
TNF-alpha	[42]	[13,35]	[8,16,23,29,35,42,43]	[8,13,21,23,29,32,37,42,43]	[29]	[8,13,21,23,26,30,32,34,35,37,38,42,43]	[29,37]	[8,23,29,32,34,38,42,43]	[8,13,16,21,23,24,29,30,32,34,35,37,42,43]
IL-1B		[13,35]	[8,11,16,23,25,29,35,43]	[8,13,28,29,32,37,43]	[29]	[8,13,26,30,31,32,34,35,36,37,43]	[29,37]	[8,29,32,34,36,43]	[8,13,16,24,29,30,31,32,34,35,37,43]
IL-6	[42]		[8,16,23,25,29,42]	[8,23,29,32,42]	[29]	[8,23,32,34,38,42]	[29]	[8,23,29,32,34,38,42]	[8,16,23,29,32,34,42]
IL-8	[42]		[16,25,29,42]	[11,29,32,42]	[29]	[32,38,42]	[29]	[29,32,42]	[16,29,32,38,42]
IL-10			[8,16,23,29]	[8,23,29,32]	[29]	[8,23,32,34,38]	[29]	[8,23,29,32,34]	[8,16,23,29,32,34,38]
IL-17A	[15]		[16]	[32]		[15,32]		[32]	[16,32]
IL-17E	[15]					[15]			
IL-7			[29]	[29,32]	[29]	[32]	[29]	[29,32]	[29,32]
IL-12/IL-12p70/IL-12A			[16,29]	[29,32]	[29]	[32,38]	[29]	[29,32,38]	[16,29,32]
IL-4			[16]	[32]		[32,38]		[32,38]	[16,32]
IL-1α			[16]			[38]		[38]	[16]
IL-1Ra			[29]	[29,32]	[29]	[32]	[29]	[29,32]	[29,32]
CCL3			[16,25,29]	[29,32]	[29]	[32]	[29]	[29,32]	[16,29,32]
CCL-11			[29]	[29]	[29]		[29]	[29]	[29]
CCL-2				[32]		[32]		[32]	[32]
CCL-4			[29]	[29,32]	[29]	[32]	[29]	[29,32]	[29,32]
PGE2						[31,36]		[36]	[22,31]
CCL-5				[32]		[32]		[32]	[32]
RANKL/sRANKL			[16,33,39,44]	[21,33,40,44]		[21,27,33,40]	[33,44]	[33,40]	[16,21,27,33,40,44]
OPG			[16,33,39]	[21,33,40]		[21,33,40]	[33]	[33,40]	[16,21,27,33,40]
RANKL:OPG									
INF-γ			[16]	[32]		[32,38]		[32,38]	[16,32]
IL-2			[16]	[32]		[32]		[32]	[16,32]
GCSF, GMCSF INF-α, IL-2r, IL-13, IL-5, IL-15, CXCL-9, CXCL-10				[32]		[32]		[32]	[32]
CRP			[16]						[16]
Biomarker	1 Day	2 Days	1 Week/ 7 Days	2 Weeks/15 days	3 Weeks	4 weeks/30 Days/1 Month	6 Weeks	8 Weeks/2 Months/60 Days	90 Days/12 Weeks/3 Months
